# Clinical trial registration was associated with lower risk of bias compared with non-registered trials among trials included in systematic reviews

**DOI:** 10.1016/j.jclinepi.2022.01.012

**Published:** 2022-01-23

**Authors:** Kristina Lindsley, Nicole Fusco, Tianjing Li, Rob Scholten, Lotty Hooft

**Affiliations:** a Julius Center for Health Sciences and Primary Care, University Medical Center Utrecht, Utrecht University, Utrecht, The Netherlands; b Cochrane Netherlands, University Medical Center Utrecht, Utrecht University, Utrecht, The Netherlands; c Xcenda, LLC, Boston, MA; d Department of Ophthalmology, School of Medicine, University of Colorado Anschutz Medical Campus, Aurora, CO

**Keywords:** Trial registration, Risk of bias, Research methods, Evidence synthesis, Systematic review, Randomized controlled trial

## Abstract

**Objective:**

To examine the association between clinical trial registration and risk of bias in clinical trials that have been included in systematic reviews. As a secondary objective, we evaluated the risk of bias among trials registered prospectively vs. retrospectively.

**Method:**

Clinical trials published in 2005 or after included in a sample of 100 Cochrane systematic reviews published from 2014–2019.

**Results:**

Of 1,177 clinical trials identified, we verified 368 (31%) had been registered, of which 135 (36.7%) were registered prospectively (i.e., before or up to 1 month after enrollment of the first participant). Across the bias domains (one bias assessment for each domain per trial), the percentage of trials at low risk ranged from 29% to 58%; unclear risk ranged from to 26% to 61% and high risk ranged from 2% to 38%. Trials that had been registered had less high or unclear risk of bias in five domains: random sequence generation (univariate risk ratio [RR] 0.69, 95% confidence interval [95% CI] 0.58–0.81), allocation concealment (RR 0.64, 95% CI 0.57–0.72), performance bias (RR 0.65, 95% CI 0.58–0.72), detection bias (RR 0.70, 95% CI 0.62–0.78), and reporting bias (RR 0.62, 95% CI 0.53–0.73). An association between clinical trial registration and high or unclear risk of attrition bias could not be demonstrated nor refuted (RR 1.02, 95% CI 0.89–1.17). It also was observed in terms of overall risk of bias, that registered trials had less high or unclear overall risk of bias than trials that had not been registered (univariate RR 0.29, 95% CI 0.19–0.46). Prospective clinical trial registration was associated with low risks of selection bias due to inadequate allocation concealment, performance bias, and detection bias compared with retrospective clinical trial registration.

**Conclusion:**

In a large sample of clinical trials included in recently published systematic reviews of interventions, clinical trial registration was associated with low risk of bias for five of the six domains examined.

## 1. Introduction

In 2004, the International Committee of Medical Journal Editors (ICMJE) published the recommendation that any clinical trial being submitted for publication should be registered in a publicly accessible clinical trial register.[1] The online clinical trial register ClinicalTrials.gov, which was made available to the public in 2000, saw a substantial increase in the number of trial registrations following ICMJE’s recommendation, and even more after the Food and Drug Administration Amendment Act of 2007,[2] which required that clinical trials used for regulatory approval of pharmaceuticals in the United States be registered.[3,4] Furthermore, the Consolidated Standards of Reporting Trials (CONSORT) guideline also includes trial registration number and name of trial registry as part of their reporting checklist.[5]

Much research has been conducted on the utility of clinical trial registration records in evidence synthesis.[6–11] A key advantage of trial registration in research, beyond the creation of a public record indicating that the trial has taken place, is that trial registry records may also serve as trial protocol repositories, establishing the intended methods and outcomes of a clinical trial before results are known. This additional source of trial information may fill in gaps about the methods and results of trials that may not make it into journal publications or conference abstracts due to space limitations and other reasons, and thus facilitate systematic reviewers in assessing the risk of bias of included trials.

Risk of bias assessment is a critical step when performing a systematic review as it provides the confidence that the review findings can be trusted and applied to health care decision making. There have many advances in the understanding of bias in clinical research, as reflected in the updated Cochrane Risk of Bias tool.[12] This study aims to evaluate the relationship between clinical trial registration and risk of bias among clinical trials included in recently published systematic reviews.

## 2. Objectives

To examine the association between trial registration and risk of bias among clinical trials included in systematic reviews of healthcare interventions. Specifically, we assessed whether clinical trials (published in 2005 and after) that were included in systematic reviews had been registered in clinical trial registers and the relationship with risk of bias (high, low, or unclear) for each domain according to the Cochrane Risk of Bias tool used by the systematic reviewers (v1; 2011).[13] Secondary objectives were to evaluate the overall risk of bias and risk of bias among trials registered prospectively vs. retrospectively.

## 3. Methods

### 3.1. Data source

This research was conducted in accordance with a protocol that included prespecified objectives, variable definitions, and analysis plan; methods for data collection have been described previously.[14] Briefly, we selected a sample of systematic reviews of intervention effectiveness from the Cochrane Musculoskeletal, Oral, Skin and Sensory (MOSS) network portfolio of reviews published from September 2014 to September 2019. Between 2019 and 2020, Cochrane began recommending using a second version of their Risk of Bias Tool (Sterne et al, 2020); thus, this research includes only reviews that used the first version for consistency of results. The MOSS network includes eight topic-specific review groups: (1) Back and Neck; ((2) Ear, Nose and Throat; (3) Eyes and Vision; (4) Musculoskeletal; (5) Oral Health; (6) Pain, Palliative and Supportive Care; (7) Skin; and (8) Wounds. From seven of the eight topic-specific review groups, we selected a random sample of 10 intervention reviews that included at least five clinical trials; we selected 30 Eyes and Vision reviews as part of the initial pilot project. Thus, we included a total of 100 Cochrane systematic reviews in our sample (references supplied in Appendix A). These 100 reviews included 2000 trials, 1177 of which were published in 2005 or after. We selected the date of 2005 based on when the ICMJE criteria for trial registration came into effect. Any trial design (e.g., parallel group trial, cross-over trial) was eligible for inclusion.

### 3.2. Data collection

Two individuals independently extracted data, including review characteristics, such as the condition under investigation, the interventions and comparisons being examined, and the number of included trials, as well as the characteristics of the trials included in each review, such as when the trial was conducted, the number of participants randomized, and whether a trial registration ID was reported by the review authors. DistillerSR (Evidence Partners) was used for data extraction. We verified all trial registration IDs provided by the review authors. When no trial registration number was reported by the review authors and the trial was published in 2005 or more recently, we first searched the original study reports. Then, if no trial registration number was provided in the reports, we searched trial registers to determine if the trial was registered. Two individuals searched ClinicalTrials.gov (www.clinicaltrials.gov) and the WHO IC-TRP (www.who.int/ictrp/en/), the two clinical trial registry databases that are endorsed by the ICMJE, using a combination of condition and intervention terms. We confirmed trial registration matching by comparing the study investigators and/or institutions and sponsors, the number of participants, the study period, and the study design. We developed an algorithm in Python (PyCharm; JetBrains s.r.o. 2020) to automatically extract the risk of bias assessments (domains and judgements of high, low, or unclear risk of bias) from an html file for each included Cochrane review. We checked the reliability of the data collected by the algorithm against the risk of bias tables in the reviews.

### 3.3. Data analysis for primary objective

We summarized review and trial level characteristics descriptively (medians, ranges, and proportions) using RStudio (R version 3.6.1) with an assumption of independence by checking that no trial was included in more than one review. Between group differences were compared using the Chi-squared test, with *P* < 0.05 indicating statistical significance.

The primary association of interest was between clinical trial registration and risk of bias among trials that were included in systematic reviews of interventions and published in 2005 or more recently (N = 1,177). The independent variable or determinant was trial registration, and the outcome was high or unclear risk of bias. Thus risk ratios (RR) greater than 1 suggest an association between clinical trial registration and high or unclear risk of bias and RRs less than 1 suggest that clinical trial registration is associated with low risk of bias.

We analyzed each of the following main risk of bias domains individually: random sequence generation, allocation concealment, performance bias, detection bias, attrition bias, and reporting bias. We employed a complete case analysis such that risk of bias domains not assessed by review authors were excluded from the analysis; however, as a mandatory requirement, few reviews did not assess all risk of bias domains. One risk of bias assessment per domain was analyzed per trial. We used the assessments as reported by the review authors regardless of study design. In cases where review authors assessed the risk of bias for multiple outcomes, the assessment of the primary review outcome was selected for that domain. In cases where review authors used variations in wording, we classified the assessment according to the appropriate risk of bias domain.

We performed univariate analysis and examined the following covariates of interest using multivariable logistic regression (glm function in RStudio): year of publication (continuous), number of participants (continuous), type of intervention (pharmaceutical vs. non-pharmaceutical), study design (parallel-group RCT vs. others), geographical region (Europe, North America, and multiregional vs others), and availability of an open access full-text publication (yes vs. no). Non-pharmaceutical interventions comprised devices, surgery, and behavioral interventions, including physiotherapy, diet, and self-care programs.

For the primary analyses, risk of bias per domain was dichotomized as high or unclear vs. low. We conducted sensitivity analyses (1) comparing high risk of bias vs. low or unclear and (2) excluding assessments of unclear risk of bias from the analysis (high vs. low risk of bias).

### 3.4. Data analysis for secondary objectives

We followed the recommendation from the Cochrane Risk of Bias tool to classify an overall risk of bias for each trial as follows: overall low risk of bias when low risk of bias was assessed for all key domains, overall unclear risk of bias when unclear risk of bias was assessed for one or more key domains, and overall high risk of bias when high risk of bias was assessed for one or more key domains.[13] We performed univariate analysis, multivariable analysis, and sensitivity analyses according the same methods as with the primary objective; however, due to the small number of studies with overall low risk of bias (“non-exposed” group), the analyses were performed using the inverse estimates.

Secondary analysis also compared the risk of bias among trials registered prospectively vs. retrospectively. Prospective registration was considered a first posting date prior to, or up to 1 month after, the date of when the first participant was enrolled. Any registration first registered more than 1 month after the date of participant enrollment was classified as retrospective registration. In the analysis prospective registration was considered the determinant and high/unclear risk of bias was the outcome.

## 4. Results

### 4.1. Characteristics of included trials

We identified 1,177 trials from a sample of 100 recently published reviews (median: 9 trials per review) from the Cochrane MOSS Network and published as of 2005, the first full calendar year in which the ICMJE recommended trial registration for publication. The median year of publication was 2010 (range 2005–2018) and the trials included 230,161 total participants (median 68 per trial). The most common study design was the randomized parallel-group trial (1036, 88%). Most trials were conducted in Asia/Pacific and Europe, followed by North America and Africa/Middle East. Half of the trials had full text reports available free of charge to the public. Clinical trial registration was found for 368 (31%) trials; of those 135 (36.7%) were registered prospectively. Of note, trial registration numbers were reported by review authors for only 180 trials; we identified the remaining 188 trial registrations by manually searching the clinical trial registers. Compared with trials with no clinical trial registration, registered trials were less likely to have been published before 2015 and more likely to include 100 or more participants, examine pharmaceutical interventions, and have an open access publication (Table 1).

### 4.2. Risk of bias of included trials

We examined each of the predefined risk of bias domains individually across all studies and for trials that were registered (n = 368) compared with trials that were not registered (n = 809). All reviews assessed random sequence generation, allocation concealment, and attrition bias. Seven reviews (71 trials, 6%) did not provide assessments for performance bias, three reviews (18 trials, 2%) did not assess detection bias, and six reviews (91 trials, 8%) did not assess reporting bias. Most review authors (94 reviews) reported that the risk of bias assessments were performed independently by at least two individuals; for five reviews it was reported only that assessments were done according to standard Cochrane methods;[15–19] and one review was conducted by a single author.[20]

**Fig. 1.**
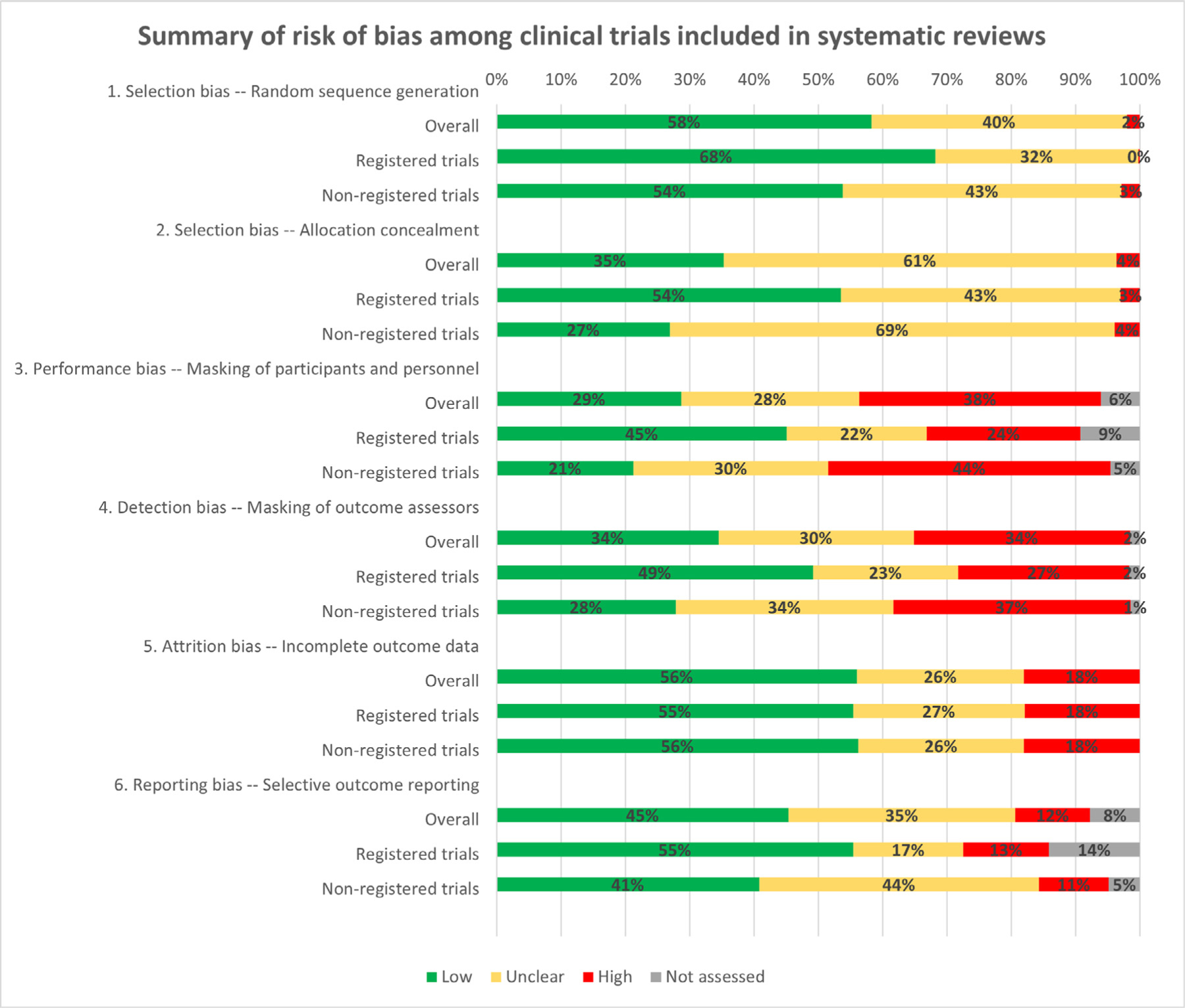
Summary of risk of bias among clinical trials included in systematic reviews. *All risk of bias domains, except for attrition bias, were significantly associated with clinical trial registration. (For interpretation of the references to color in this figure legend, the reader is referred to the Web version of this article.)

Overall, three domains were assessed as low risk for 45% or more trials: random sequence generation, attrition bias, and reporting bias (Fig. 1). The majority of studies were assessed as having unclear risk of bias for allocation concealment (61%). Performance and detection biases were assessed as high risk for more than one third of trials (38% and 34%, respectively). In terms of overall risk of bias, 74 trials (6%) were at low risk, 402 trials (34%) were at unclear risk, and 701 (60%) were at high risk.

### 4.3. Association of clinical trial registration and risk of bias

All risk of bias domains, with the exception of attrition bias, were significantly associated with clinical trial registration in that registered trials were more likely to have been assessed as having low risk of bias, in both univariate and multivariable analyses (Table 2). The direction of association changed for one risk of bias domain in sensitivity analysis: grouping unclear with low risk of reporting bias resulted with trial registration favoring a high risk of bias, most likely a result of the smaller proportion of unclear trials in the registered group (17%) than the unregistered group (44%). Similarly, in sensitivity analysis excluding unclear risk, no association between clinical trial registration and risk of reporting bias was observed. For all other domains, excluding unclear assessments strengthened the associations. Primary, multivariable, and sensitivity analyses suggest evidence of no association between clinical trial registration and risk of attrition bias.

### 4.4. Secondary analysis Association of clinical trial registration and overall risk of bias

Of 368 registered trials, 45 (12%) were at overall low risk, 141 trials (38%) were at overall unclear risk, and 182 (49%) were at overall high risk. Of 809 trials that were not registered, 29 (4%) were at overall low risk, 261 trials (32%) were at overall unclear risk, and 519 (64%) were at overall high risk. The analyses suggest that clinical trial registration may be associated with overall low risk bias as observed with univariate, multivariable, and sensitivity analyses (Table 3).

### 4.5. Secondary analysis – Association of prospective or retrospective clinical trial registration and risk of bias

Of 368 registered trials, 135 (36.7%) were registered prospectively and 233 (63.3%) retrospectively. Secondary analyses suggest that prospective clinical trial registration may be associated with low risks of selection bias from inadequate allocation concealment, performance bias, and detection bias compared with retrospective clinical trial registration (Table 4). The association of prospective clinical trial registration also favored low risks of selection bias due to inadequate random sequence generation and reporting bias, but these were not statistically significant. As with the primary analyses, no association was observed with attrition bias, although the confidence interval was imprecise (95% CI 0.85 to 1.36).

## 5. Discussion

### 5.1. Summary of main findings

Among a large sample of clinical trials included in recently published (2015–2019) systematic reviews of interventions, this study found that clinical trial registration was associated with low risk of bias for all bias domains examined except for attrition bias, and for overall risk of bias. These findings were consistent using both univariate and multivariable regression models. For three bias domains – random sequence generation, performance bias, and detection bias – grouping unclear risk with low risk or excluding trials with unclear risk altogether did not impact the direction or significance of the associations. Evidence of no association between clinical trial registration and attrition bias was observed; however, imprecise estimates preclude a definitive conclusion of no association.

Comparing prospectively vs. retrospectively registered trials, prospectively registered trials were more likely to have low risks of selection bias due to inadequate allocation concealment, performance bias, and detection bias; no associations were noted for selection bias due to inadequate random sequence generation, attrition bias, or reporting bias; however, imprecise estimates preclude a definitive conclusion for these domains.

Our findings are in line with prior research investigating clinical trial registration and risk of bias. In a study of fertility treatment trials, 44% of 693 randomized controlled trials published between 2010 and 2014 had been registered and significant differences were observed between registered and non-registered trials for random sequence generation, allocation concealment, and selective outcome reporting.[10] Similarly, in a study of randomized controlled trials conducted in Latin America and the Caribbean and published in 2010, 17% of 526 trials had been registered in ICTRP, of which registered trials were a lower risk of overall bias than non-registered trials.[21] Because trials may be initiated for reasons other than regulatory approval or publication, examining trials included in systematic reviews may shed light more directly on the impact to evidence-based decision-making.

### 5.2. Methods for minimizing bias in clinical trials and ascertaining the impact of potential bias

Although not unexpected, clinical trial registration was associated with low risk of bias for many domains. The causes of these associations are unclear, but they could be influenced by the review authors having additional sources of information when assessing risk of bias and improved reporting of methods (such as compliance with CONSORT recommendations). It could also be that registered trials may be more likely to involve a multidisciplinary team of investigators who are aware of both methods for minimizing the risks of bias during the conduct of the trial and standard trial registration and reporting requirements.

By definition, many aspects of the design and conduct of an experimental study are directly controlled by the investigators. With respect to clinical trials, how the randomization sequence is generated, how allocation of participants is concealed, whether blinding is done, and how and which outcomes are reported are fully under to the control of investigators from the protocol stage and throughout the clinical trial lifecycle. All these methods are encompassed within the risk of bias domains that were associated with clinical trial registration in this study. For the remaining risk of bias domain examined – attrition bias – the association with clinical trial registration was inconclusive. As with all bias domains, attrition bias involves multiple factors; however, missing data, a key contributor to attrition bias, cannot be completely controlled. Although trialists can apply methods aimed at preventing participant attrition, such as compensating patients, using a run-in period, or employing a flexible treatment and follow-up schedule,[22] some reasons for missing data are outside the hands of the investigators, such as death, participants missing follow-up visits, or participants withdrawing consent. Thus, given that study attrition cannot always be controlled, the presence of missing data could be distributed evenly across studies to explain why the proportion of trials with unclear and high risk of attrition bias were the same regardless of trial registration status.

In addition to research dedicated to reducing missing data, much work has been put into improving the quality of clinical trials overall, especially with respect to the transparent reporting of trial methods and findings. In our sample of trials, all domains had a high percentage of trials assessed at high or unclear risk of bias (42%−65%). Although these data are limited to the clinical topic areas covered by the Cochrane MOSS network, prior research has reported similar percentages of high or unclear risk of bias across many different clinical areas.[23,24] It is important to note that risk of bias assessments are driven by two factors – the reporting of methods and the actual methods – and interpretation of unclear or high risk may conflate the two. Another possibility for the high number of unclear and high risk of bias assessments could be the misinterpretation of the first version of the Cochrane Risk of Bias tool.[25] Reviewers could complete their assessment driven by single, strict yes/no responses (e.g., Were any participants lost to follow-up?) and not necessarily consider how these factors would influence (i.e., bias) the effect estimates. The second version of the Risk of Bias tool,[12] which was incorporated in the 2020 update of the Cochrane Handbook,[26] addresses this issue by incorporating signaling questions for each domain and applying an algorithm to help reviewers navigate through their assessments. Study design specific versions (e.g., cross-over trials, cluster-randomized trials) also have been developed for the second version. As uptake of the new tool enters the evidence synthesis ecosystem, it will be interesting to see if the proportion of studies with unclear risk of bias assessments decreases.

### 5.3. The state of clinical trial registration and the evidence synthesis ecosystem

It has been more than 15 years since the ICMJE recommended that journals publish manuscripts of trial results only when the trial had been registered in a public trials registry. Although there was a trend of improved registration in more recent years, the overall number of registered trials in our sample was low (31% overall and 38% since 2010). Even more, of registered trials, only 37% had been registered prospectively. Other studies examining trends in clinical trial registration have also reported low rates (50% or less) of prospective trial registration.[27–30] It is important to note that the trials included in this study were identified from recently published systematic reviews of interventions (2014–2019), and thus impact current day evidence-based decision making.

The two bias domains with the largest percentage difference in unclear assessments between registered and non-registered trials were allocation concealment and reporting bias. Sensitivity analysis grouping unclear with low risk of bias impacted allocation concealment and reporting bias; excluding unclear risk of bias impacted only reporting bias. Overall, allocation concealment had the highest percentage of unclear risk of bias (61%). Currently, allocation concealment is not an explicit data element captured in the clinical trial registration record; however, it is an item on the CONSORT checklist.

A major advantage of clinical trial registries is the opportunity to compare the planned outcomes in the trial registration record with the outcomes reported in the trial publications. Even when trials had been registered retrospectively, more than one-third had issues with selective outcome reporting. In the updated Cochrane Risk of bias tool, selective outcome reporting has been replaced by assessing the bias in selection of the reported results and the assessment of selective outcome reporting is recommended to be done for the review level rather than at the trial level.[12] As switching of clinical trial outcomes remains problematic in the published literature,[31,32] the clinical trial registration record is a useful resource to identify both potential reporting bias and bias in the selection of the reported results when trials have been registered.

Also notable was that more than half of the trial registration numbers in our sample were not cited by the systematic review authors as recommended by the Methodological Expectations of Cochrane Intervention Reviews (MECIR Standards);[33] we identified 51% of trial registrations by manually searching the clinical trial registers. We also observed that, when reported, the trial registration numbers were reported in various places across reviews—most frequently in the table of characteristics of included studies or the risk of bias tables, and sometimes in the main text or as a reference to the study. It is uncertain the extent that trial registries, if searched at all, are being utilized by review authors and incorporated into the evidence ecosystem.

## 6. Conclusions and implications for research

This study found that clinical trial registration was associated with low risk of bias for five of the six domains examined, using both univariate and multivariable regression models, for a large sample of clinical trials included in systematic reviews of interventions within eight clinical topic areas. In addition to following best practice standards for registering trials prospectively, trialists should also take care to implement, and clearly report, methods for minimizing the risk of bias. Systematic reviewers should also follow guidelines (Cochrane, PRISMA 2020) for incorporating searches of the clinical trial registries and employing trial registry records when assessing the study’s risk of bias, especially as relates to selective outcome reporting and publication bias. In systematic reviews with meta-analysis, trial registration could serve as a relevant single variable for conducting sensitivity analysis to examine the impact on results.

## Supplementary Material

1

## Figures and Tables

**Table 1.** Characteristics of included trials (n = 1,177)

Trial characteristics	Total trials n = 1,177	Registered trials n = 368 (31%)	Non-registered trials n = 809 (69%)
Date of publication, median (range)	2010 (2005–2018)	2011 (2005–2018)	2010 (2005–2018)
Date of publication, number (%)*			
Published 2005 to before 2010	495	107 (22%)	388 (78%)
Published 2010 to before 2015	565	201 (36%)	364 (64%)
Published 2015 to before 2019	117	60 (51%)	57 (49%)
Trial participants, total (median per trial)	185,691 (68)	105,192 (120)	80,499 (60)
Trial participants, number (%)*			
Less than 100 participants	751	158 (21%)	593 (79%)
100 or more participants	426	210 (49%)	216 (51%)
Clinical topic area, number (%)*			
Back and neck	141	36 (26%)	105 (74%)
Ear, nose, and throat	53	14 (26%)	39 (74%)
Eyes and vision	330	100 (30%)	230 (70%)
Musculoskeletal	130	56 (43%)	74 (57%)
Oral health	98	15 (15%)	83 (85%)
Pain, palliative and supportive care	144	78 (54%)	66 (46%)
Skin	209	49 (23%)	160 (77%)
Wounds	72	20 (28%)	52 (72%)
Review intervention type, number (%)*			
Pharmaceutical	599	229 (38%)	370 (62%)
Non-pharmaceutical**	578	139 (24%)	439 (76%)
Trial design, number (%)*			
Parallel-group randomized trial	1036	339 (33%)	697 (67%)
Cluster randomized trial	4	0	4 (100%)
Cross-over randomized trial	31	10 (32%)	21 (68%)
Within-person randomized trial	94	19 (20%)	75 (80%)
Quasi-randomized trial or unclear	12	0	12 (100%)
Geographic region, number (%)*			
Africa/Middle East	182	29 (16%)	153 (84%)
Asia/Pacific	342	57 (17%)	285 (83%)
Europe	333	106 (32%)	227 (68%)
North America	193	101 (52%)	92 (48%)
South America	56	20 (36%)	36 (64%)
Multiple regions	69	55 (80%)	14 (20%)
Not reported	2	0	2 (100%)
Full text report available free of charge, number (%)			
Yes	583	216 (37%)	367 (63%)
No	594	152 (26%)	442 (74%)

* Chi-square test *P* < 0.005 comparing registered vs. non-registered trials

** Non-pharmaceutical interventions comprised devices, surgery, and behavioral interventions, including physiotherapy, diet, and self-care programs

**Table 2.** Risk ratios (RRs) for the presence of risk of bias of having been registered vs. not having been registered

Random sequence generation (selection bias)	Number	RR* (95% CI)
Primary, univariate analysis (high/unclear vs low ROB)	1,177	0.69 (0.58–0.81)
Multivariable analysis** (high/unclear vs low ROB)	1,177	0.71 (0.53–0.95)
Sensitivity analysis 1 (high vs low/unclear ROB)	1,177	0.10 (0.01–0.70)
Sensitivity analysis 2 (high vs low ROB)	710	0.08 (0.01–0.58)
**Allocation concealment (selection bias)**	**Number**	**RR (95% CI)**

Primary, univariate analysis (high/unclear vs low ROB)	1,177	0.64 (0.57–0.72)
Multivariable analysis** (high/unclear vs low ROB)	1,177	0.45 (0.34–0.61)
Sensitivity analysis 1 (high vs low/unclear ROB)	1,177	0.76 (0.39–1.48)
Sensitivity analysis 2 (high vs low ROB)	458	0.41 (0.21–0.80)
**Blinding of participants and personnel (performance bias)**	**Number**	**RR (95% CI)**

Primary, univariate analysis (high/unclear vs low ROB)	1,106	0.65 (0.58–0.72)
Multivariable analysis** (high/unclear vs low ROB)	1,106	0.39 (0.28–0.53)
Sensitivity analysis 1 (high vs low/unclear ROB)	1,106	0.57 (0.47–0.70)
Sensitivity analysis 2 (high vs low ROB)	781	0.51 (0.43–0.62)
**Blinding of outcome assessment (detection bias)**	**Number**	**RR (95% CI)**

Primary, univariate analysis (high/unclear vs low ROB)	1,159	0.70 (0.62–0.78)
Multivariable analysis** (high/unclear vs low ROB)	1,159	0.53 (0.40–0.72)
Sensitivity analysis 1 (high vs low/unclear ROB)	1,159	0.72 (0.60–0.88)
Sensitivity analysis 2 (high vs low ROB)	802	0.62 (0.52–0.74)
**Incomplete outcome data (attrition bias)**	**Number**	**RR (95% CI)**

Primary, univariate analysis (high/unclear vs low ROB)	1,177	1.02 (0.89–1.17)
Multivariable analysis** (high/unclear vs low ROB)	1,177	1.11 (0.84–1.47)
Sensitivity analysis 1 (high vs low/unclear ROB)	1,177	0.99 (0.76–1.29)
Sensitivity analysis 2 (high vs low ROB)	871	1.01 (0.78–1.30)
**Selective reporting (reporting bias)**	**Number**	**RR (95% CI)**

Primary, univariate analysis (high/unclear vs low ROB)	1,086	0.62 (0.53–0.73)
Multivariable analysis** (high/unclear vs low ROB)	1,086	0.45 (0.34–0.61)
Sensitivity analysis 1 (high vs low/unclear ROB)	1,086	1.36 (0.98–1.88)
Sensitivity analysis 2 (high vs low ROB)	671	0.92 (0.67–1.26)

95% CI, 95% confidence interval; ROB, risk of bias; RR, risk ratio

* High or unclear risk of bias compared with low risk; RR *<* 1 indicates low risk associated with trial registration, RR *>* 1 indicates high/unclear risk associated with trial registration

** Full multivariable model included the following: year of publication (continuous), number of participants (continuous), type of intervention (pharmaceutical vs non-pharmaceutical), study design (parallel-group RCT vs others), geographical region (Europe, North America, and multiregional vs others), and availability of an open access full-text publication (yes vs no); all sensitivity analyses are univariate.

**Table 3.** Risk ratios (RRs) for the presence of overall risk of bias of having been registered vs. not having been registered

Overall risk of bias	Number	RR* (95% CI)
Primary, univariate analysis (high/unclear vs low ROB)	1,177	0.29 (0.19–0.46)
Multivariable analysis** (high/unclear vs low ROB)	1,177	0.31 (0.18–0.54)
Sensitivity analysis 1 (high vs low/unclear ROB)	1,177	0.71 (0.62–0.81)
Sensitivity analysis 2 (high vs low ROB)	775	0.27 (0.17–0.41)

95% CI, 95% confidence interval; ROB, risk of bias; RR, risk ratio

* High or unclear risk of bias compared with low risk; RR *<* 1 indicates low risk associated with trial registration, RR *>* 1 indicates high/unclear risk associated with trial registration

** Full multivariable model included the following: year of publication (continuous), number of participants (continuous), type of intervention (pharmaceutical vs non-pharmaceutical), study design (parallel-group RCT vs others), geographical region (Europe, North America, and multiregional vs others), and availability of an open access full-text publication (yes vs no); all sensitivity analyses are univariate.

**Table 4.** Secondary analyses: Risk ratios for the presence of high or unclear risk of bias of prospective vs. retrospective registration

Registered trials (n = 368)	High or unclear ROB, proportion (%)		
	Prospective	Retrospective	RR* (95% CI)
Random sequence generation (selection bias)	39/135 (29%)	78/233 (33%)	0.86 (0.63–1.19)
Allocation concealment (selection bias)	48/135 (36%)	123/233 (53%)	0.67 (0.52–0.87)
Blinding of participants and personnel (performance bias)	43/126 (34%)	125/208 (60%)	0.57 (0.43–0.74)
Blinding of outcome assessment (detection bias)	52/133 (39%)	129/229 (56%)	0.69 (0.55–0.88)
Incomplete outcome data (attrition bias)	63/135 (47%)	101/233 (43%)	1.08 (0.85–1.36)
Selective reporting (reporting bias)	33/101 (33%)	79/215 (37%)	0.89 (0.64–1.24)

95% CI: 95% confidence interval (bold indicates the 95% CI does not cross null); ROB: risk of bias; RR: risk ratio

* High or unclear risk of bias compared with low risk; RR *<* 1 indicates low risk associated with prospective trial registration, RR *>* 1 indicates high/unclear risk associated with prospective trial registration

## What is new?

### Key findings:

- Less than one-third of trials included in recent systematic reviews had been registered; less than one-third of registered trials were registered prospectively.
- Clinical trial registration was associated with low risk of bias for five domains: random sequence generation, allocation concealment, performance bias, detection bias, and reporting bias

### What this adds to what is known

- The design, conduct, and reporting of clinical trials are interwoven and both are necessary for adequately assessing the quality of the evidence.

### What is the implication, what should change now

- Clinical trial investigators should include trial registration as part of the process of conducting a trial
- Multi-disciplinary teams should be involved to address both content and methodological aspects of designing and conducting trials
