# Supplementary material for: Clinical trial registration was associated with lower risk of bias compared with non-registered trials among trials included in systematic reviews
Source: J Clin Epidemiol. Author manuscript; Available in PMC 2023 May 1. (PMC9875740; doi:10.1016/j.jclinepi.2022.01.012)
Supplement: 1 [file NIHMS1858699-supplement-1.docx]

References for 100 included Cochrane reviews

1. Abudou M, Wu T, Evans JR, Chen X. Immunosuppressants for the prophylaxis of corneal graft rejection after penetrating keratoplasty. Cochrane Database of Systematic Reviews 2015(8).

2. Albuquerque JVD, Andriolo BNG, Vasconcellos MRA, Civile VT, Lyddiatt A, Trevisani VFM. Interventions for morphea. Cochrane Database of Systematic Reviews 2019(7).

3. Almeida C, Choy EHS, Hewlett S, Kirwan JR, Cramp F, Chalder T, et al. Biologic interventions for fatigue in rheumatoid arthritis. Cochrane Database of Systematic Reviews 2016(6).

4. Barbaric J, Abbott R, Posadzki P, Car M, Gunn LH, Layton AM, et al. Light therapies for acne. Cochrane Database of Systematic Reviews 2016(9).

5. Bartels EM, Juhl CB, Christensen R, Hagen KB, Danneskiold‐Samsøe B, Dagfinrud H, et al. Aquatic exercise for the treatment of knee and hip osteoarthritis. Cochrane Database of Systematic Reviews 2016(3).

6. Batista K, Thiruvenkatachari B, Harrison JE, O'Brien KD. Orthodontic treatment for prominent upper front teeth (class ii malocclusion) in children and adolescents. Cochrane Database of Systematic Reviews 2018(3).

7. Bidonde J, Busch AJ, Webber SC, Schachter CL, Danyliw A, Overend TJ, et al. Aquatic exercise training for fibromyalgia. Cochrane Database of Systematic Reviews 2014(10).

8. Breederveld RS, Tuinebreijer WE. Recombinant human growth hormone for treating burns and donor sites. Cochrane Database of Systematic Reviews 2014(9).

9. Burton M, Habtamu E, Ho D, Gower EW. Interventions for trachoma trichiasis. Cochrane Database of Systematic Reviews 2015(11).

10. Cabourne E, Clarke JCK, Schlottmann PG, Evans JR. Mitomycin c versus 5‐fluorouracil for wound healing in glaucoma surgery. Cochrane Database of Systematic Reviews 2015(11).

11. Candy B, Jones L, Vickerstaff V, Tookman A, King M. Interventions for sexual dysfunction following treatments for cancer in women. Cochrane Database of Systematic Reviews 2016(2).

12. Cao H, Yang G, Wang Y, Liu JP, Smith CA, Luo H, et al. Complementary therapies for acne vulgaris. Cochrane Database of Systematic Reviews 2015(1).

13. Chang BA, Thamboo A, Burton MJ, Diamond C, Nunez DA. Needle aspiration versus incision and drainage for the treatment of peritonsillar abscess. Cochrane Database of Systematic Reviews 2016(12).

14. Chen X, Jiang X, Yang M, González U, Lin X, Hua X, et al. Systemic antifungal therapy for tinea capitis in children. Cochrane Database of Systematic Reviews 2016(5).

15. Chong LY, Head K, Hopkins C, Philpott C, Schilder AGM, Burton MJ. Intranasal steroids versus placebo or no intervention for chronic rhinosinusitis. Cochrane Database of Systematic Reviews 2016(4).

16. Christoffers WA, Coenraads PJ, Svensson Å, Diepgen TL, Dickinson‐Blok JL, Xia J, et al. Interventions for hand eczema. Cochrane Database of Systematic Reviews 2019(4).

17. Clearfield E, Muthappan V, Wang X, Kuo IC. Conjunctival autograft for pterygium. Cochrane Database of Systematic Reviews 2016(2).

18. Clive AO, Jones HE, Bhatnagar R, Preston NJ, Maskell N. Interventions for the management of malignant pleural effusions: A network meta‐analysis. Cochrane Database of Systematic Reviews 2016(5).

19. Cullum N, Liu Z. Therapeutic ultrasound for venous leg ulcers. Cochrane Database of Systematic Reviews 2017(5).

20. Dalal A, Eskin‐Schwartz M, Mimouni D, Ray S, Days W, Hodak E, et al. Interventions for the prevention of recurrent erysipelas and cellulitis. Cochrane Database of Systematic Reviews 2017(6).

21. Day AC, Gore DM, Bunce C, Evans JR. Laser‐assisted cataract surgery versus standard ultrasound phacoemulsification cataract surgery. Cochrane Database of Systematic Reviews 2016(7).

22. de Silva SR, Evans JR, Kirthi V, Ziaei M, Leyland M. Multifocal versus monofocal intraocular lenses after cataract extraction. Cochrane Database of Systematic Reviews 2016(12).

23. Derry S, Bell RF, Straube S, Wiffen PJ, Aldington D, Moore RA. Pregabalin for neuropathic pain in adults. Cochrane Database of Systematic Reviews 2019(1).

24. Derry S, Wiffen PJ, Moore RA, Bendtsen L. Ibuprofen for acute treatment of episodic tension‐type headache in adults. Cochrane Database of Systematic Reviews 2015(7).

25. Dorri M, Martinez‐Zapata MJ, Walsh T, Marinho VCC, Sheiham A, Zaror C. Atraumatic restorative treatment versus conventional restorative treatment for managing dental caries. Cochrane Database of Systematic Reviews 2017(12).

26. Downie LE, Busija L, Keller PR. Blue‐light filtering intraocular lenses (iols) for protecting macular health. Cochrane Database of Systematic Reviews 2018(5).

27. Enthoven WTM, Roelofs P, Deyo RA, van Tulder MW, Koes BW. Non‐steroidal anti‐inflammatory drugs for chronic low back pain. Cochrane Database of Systematic Reviews 2016(2).

28. Ervin AM, Law A, Pucker AD. Punctal occlusion for dry eye syndrome. Cochrane Database of Systematic Reviews 2017(6).

29. Evans JR, Lawrenson JG. Antioxidant vitamin and mineral supplements for slowing the progression of age‐related macular degeneration. Cochrane Database of Systematic Reviews 2017(7).

30. Ferguson MA, Kitterick PT, Chong LY, Edmondson‐Jones M, Barker F, Hoare DJ. Hearing aids for mild to moderate hearing loss in adults. Cochrane Database of Systematic Reviews 2017(9).

31. Fidahic M, Jelicic Kadic A, Radic M, Puljak L. Celecoxib for rheumatoid arthritis. Cochrane Database of Systematic Reviews 2017(6).

32. FlorCruz NV, Evans JR. Medical interventions for fungal keratitis. Cochrane Database of Systematic Reviews 2015(4).

33. Foo VHX, Htoon HM, Welsbie DS, Perera SA. Aqueous shunts with mitomycin c versus aqueous shunts alone for glaucoma. Cochrane Database of Systematic Reviews 2019(4).

34. Fransen M, McConnell S, Harmer AR, Van der Esch M, Simic M, Bennell KL. Exercise for osteoarthritis of the knee. Cochrane Database of Systematic Reviews 2015(1).

35. Fujiwara T, Kuriyama A, Kato Y, Fukuoka T, Ota E. Perioperative local anaesthesia for reducing pain following septal surgery. Cochrane Database of Systematic Reviews 2018(8).

36. Furlan AD, Giraldo M, Baskwill A, Irvin E, Imamura M. Massage for low‐back pain. Cochrane Database of Systematic Reviews 2015(9).

37. George R, Sundararaj JJ, Govindaraj R, Chacko AG, Tharyan P. Interventions for the treatment of metastatic extradural spinal cord compression in adults. Cochrane Database of Systematic Reviews 2015(9).

38. Gibson W, Wand BM, O'Connell NE. Transcutaneous electrical nerve stimulation (tens) for neuropathic pain in adults. Cochrane Database of Systematic Reviews 2017(9).

39. Gower EW, Lindsley K, Tulenko SE, Nanji AA, Leyngold I, McDonnell PJ. Perioperative antibiotics for prevention of acute endophthalmitis after cataract surgery. Cochrane Database of Systematic Reviews 2017(2).

40. Head K, Chong LY, Hopkins C, Philpott C, Burton MJ, Schilder AGM. Short‐course oral steroids alone for chronic rhinosinusitis. Cochrane Database of Systematic Reviews 2016(4).

41. Jüni P, Hari R, Rutjes AWS, Fischer R, Silletta MG, Reichenbach S, et al. Intra‐articular corticosteroid for knee osteoarthritis. Cochrane Database of Systematic Reviews 2015(10).

42. Kamper SJ, Apeldoorn AT, Chiarotto A, Smeets RJ, Ostelo R, Guzman J, et al. Multidisciplinary biopsychosocial rehabilitation for chronic low back pain. Cochrane Database of Systematic Reviews 2014(9).

43. Kumbargere Nagraj S, George RP, Shetty N, Levenson D, Ferraiolo DM, Shrestha A. Interventions for managing taste disturbances. Cochrane Database of Systematic Reviews 2017(12).

44. Lai NM, Lai NA, O'Riordan E, Chaiyakunapruk N, Taylor JE, Tan K. Skin antisepsis for reducing central venous catheter‐related infections. Cochrane Database of Systematic Reviews 2016(7).

45. Lam FC, Chia SN, Lee RMH. Macular grid laser photocoagulation for branch retinal vein occlusion. Cochrane Database of Systematic Reviews 2015(5).

46. Le JT, Bicket AK, Wang L, Li T. Ab interno trabecular bypass surgery with istent for open‐angle glaucoma. Cochrane Database of Systematic Reviews 2019(3).

47. Li T, Qureshi R, Taylor K. Conventional occlusion versus pharmacologic penalization for amblyopia. Cochrane Database of Systematic Reviews 2019(8).

48. Lim BX, Lim CHL, Lim DK, Evans JR, Bunce C, Wormald R. Prophylactic non‐steroidal anti‐inflammatory drugs for the prevention of macular oedema after cataract surgery. Cochrane Database of Systematic Reviews 2016(11).

49. Lim CHL, Turner A, Lim BX. Patching for corneal abrasion. Cochrane Database of Systematic Reviews 2016(7).

50. Linde K, Allais G, Brinkhaus B, Fei Y, Mehring M, Vertosick EA, et al. Acupuncture for the prevention of episodic migraine. Cochrane Database of Systematic Reviews 2016(6).

51. Liu Z, Dumville JC, Hinchliffe RJ, Cullum N, Game F, Stubbs N, et al. Negative pressure wound therapy for treating foot wounds in people with diabetes mellitus. Cochrane Database of Systematic Reviews 2018(10).

52. Ma X, Li C, Jia L, Wang Y, Liu W, Zhou X, et al. Materials for retrograde filling in root canal therapy. Cochrane Database of Systematic Reviews 2016(12).

53. Maas ET, Ostelo R, Niemisto L, Jousimaa J, Hurri H, Malmivaara A, et al. Radiofrequency denervation for chronic low back pain. Cochrane Database of Systematic Reviews 2015(10).

54. Machado GC, Ferreira PH, Yoo RIJ, Harris IA, Pinheiro MB, Koes BW, et al. Surgical options for lumbar spinal stenosis. Cochrane Database of Systematic Reviews 2016(11).

55. Manfredi M, Figini L, Gagliani M, Lodi G. Single versus multiple visits for endodontic treatment of permanent teeth. Cochrane Database of Systematic Reviews 2016(12).

56. Marinho VCC, Worthington HV, Walsh T, Chong LY. Fluoride gels for preventing dental caries in children and adolescents. Cochrane Database of Systematic Reviews 2015(6).

57. Martinez‐Zapata MJ, Martí‐Carvajal AJ, Solà I, Pijoán JI, Buil‐Calvo JA, Cordero JA, et al. Anti‐vascular endothelial growth factor for proliferative diabetic retinopathy. Cochrane Database of Systematic Reviews 2014(11).

58. Matterne U, Böhmer MM, Weisshaar E, Jupiter A, Carter B, Apfelbacher CJ. Oral h1 antihistamines as ‘add‐on’ therapy to topical treatment for eczema. Cochrane Database of Systematic Reviews 2019(1).

59. Moja L, Lucenteforte E, Kwag KH, Bertele V, Campomori A, Chakravarthy U, et al. Systemic safety of bevacizumab versus ranibizumab for neovascular age‐related macular degeneration. Cochrane Database of Systematic Reviews 2014(9).

60. Monk AB, Harrison JE, Worthington HV, Teague A. Pharmacological interventions for pain relief during orthodontic treatment. Cochrane Database of Systematic Reviews 2017(11).

61. Monticone M, Cedraschi C, Ambrosini E, Rocca B, Fiorentini R, Restelli M, et al. Cognitive‐behavioural treatment for subacute and chronic neck pain. Cochrane Database of Systematic Reviews 2015(5).

62. Moore ZEH, Webster J. Dressings and topical agents for preventing pressure ulcers. Cochrane Database of Systematic Reviews 2018(12).

63. Nankervis H, Pynn EV, Boyle RJ, Rushton L, Williams HC, Hewson DM, et al. House dust mite reduction and avoidance measures for treating eczema. Cochrane Database of Systematic Reviews 2015(1).

64. Norman G, Dumville JC, Mohapatra DP, Owens GL, Crosbie EJ. Antibiotics and antiseptics for surgical wounds healing by secondary intention. Cochrane Database of Systematic Reviews 2016(3).

65. Page MJ, Green S, Mrocki MA, Surace SJ, Deitch J, McBain B, et al. Electrotherapy modalities for rotator cuff disease. Cochrane Database of Systematic Reviews 2016(6).

66. Pan Q, Angelina A, Marrone M, Stark WJ, Akpek EK. Autologous serum eye drops for dry eye. Cochrane Database of Systematic Reviews 2017(2).

67. Payne AGT, Alsabeeha NHM, Atieh MA, Esposito M, Ma S, Anas El‐Wegoud M. Interventions for replacing missing teeth: Attachment systems for implant overdentures in edentulous jaws. Cochrane Database of Systematic Reviews 2018(10).

68. Perry A, Lee SH, Cotton S, Kennedy C. Therapeutic exercises for affecting post‐treatment swallowing in people treated for advanced‐stage head and neck cancers. Cochrane Database of Systematic Reviews 2016(8).

69. Poggio CE, Ercoli C, Rispoli L, Maiorana C, Esposito M. Metal‐free materials for fixed prosthodontic restorations. Cochrane Database of Systematic Reviews 2017(12).

70. Rasmussen‐Barr E, Held U, Grooten WJA, Roelofs P, Koes BW, van Tulder MW, et al. Non‐steroidal anti‐inflammatory drugs for sciatica. Cochrane Database of Systematic Reviews 2016(10).

71. Reinar LM, Forsetlund L, Lehman LF, Brurberg KG. Interventions for ulceration and other skin changes caused by nerve damage in leprosy. Cochrane Database of Systematic Reviews 2019(7).

72. Rirash F, Tingey PC, Harding SE, Maxwell LJ, Tanjong Ghogomu E, Wells GA, et al. Calcium channel blockers for primary and secondary raynaud's phenomenon. Cochrane Database of Systematic Reviews 2017(12).

73. Rowe FJ, Hanna K, Evans JR, Noonan CP, Garcia‐Finana M, Dodridge CS, et al. Interventions for eye movement disorders due to acquired brain injury. Cochrane Database of Systematic Reviews 2018(3).

74. Salehi M, Wenick AS, Law HA, Evans JR, Gehlbach P. Interventions for central serous chorioretinopathy: A network meta‐analysis. Cochrane Database of Systematic Reviews 2015(12).

75. Saragiotto BT, Maher CG, Yamato TP, Costa LOP, Menezes Costa LC, Ostelo R, et al. Motor control exercise for chronic non‐specific low‐back pain. Cochrane Database of Systematic Reviews 2016(1).

76. Schenkel AB, Veitz‐Keenan A. Dental cavity liners for class i and class ii resin‐based composite restorations. Cochrane Database of Systematic Reviews 2019(3).

77. Selva Olid A, Solà I, Barajas‐Nava LA, Gianneo OD, Bonfill Cosp X, Lipsky BA. Systemic antibiotics for treating diabetic foot infections. Cochrane Database of Systematic Reviews 2015(9).

78. Sereda M, Xia J, El Refaie A, Hall DA, Hoare DJ. Sound therapy (using amplification devices and/or sound generators) for tinnitus. Cochrane Database of Systematic Reviews 2018(12).

79. Smith JM, Steel DHW. Anti‐vascular endothelial growth factor for prevention of postoperative vitreous cavity haemorrhage after vitrectomy for proliferative diabetic retinopathy. Cochrane Database of Systematic Reviews 2015(8).

80. Straube C, Derry S, Jackson KC, Wiffen PJ, Bell RF, Strassels S, et al. Codeine, alone and with paracetamol (acetaminophen), for cancer pain. Cochrane Database of Systematic Reviews 2014(9).

81. Tseng VL, Coleman AL, Chang MY, Caprioli J. Aqueous shunts for glaucoma. Cochrane Database of Systematic Reviews 2017(7).

82. van Zuuren EJ, Fedorowicz Z, Christensen R, Lavrijsen APM, Arents BWM. Emollients and moisturisers for eczema. Cochrane Database of Systematic Reviews 2017(2).

83. Venekamp RP, Burton MJ, van Dongen TMA, van der Heijden GJ, van Zon A, Schilder AGM. Antibiotics for otitis media with effusion in children. Cochrane Database of Systematic Reviews 2016(6).

84. Venekamp RP, Mick P, Schilder AGM, Nunez DA. Grommets (ventilation tubes) for recurrent acute otitis media in children. Cochrane Database of Systematic Reviews 2018(5).

85. Virgili G, Acosta R, Bentley SA, Giacomelli G, Allcock C, Evans JR. Reading aids for adults with low vision. Cochrane Database of Systematic Reviews 2018(4).

86. Wang X, Khan R, Coleman A. Device‐modified trabeculectomy for glaucoma. Cochrane Database of Systematic Reviews 2015(12).

87. Watson SL, Leung V. Interventions for recurrent corneal erosions. Cochrane Database of Systematic Reviews 2018(7).

88. Webster J, Alghamdi A. Use of plastic adhesive drapes during surgery for preventing surgical site infection. Cochrane Database of Systematic Reviews 2015(4).

89. Wegner I, Hall DA, Smit AL, McFerran D, Stegeman I. Betahistine for tinnitus. Cochrane Database of Systematic Reviews 2018(12).

90. Welsch P, Üçeyler N, Klose P, Walitt B, Häuser W. Serotonin and noradrenaline reuptake inhibitors (snris) for fibromyalgia. Cochrane Database of Systematic Reviews 2018(2).

91. Westby MJ, Norman G, Dumville JC, Stubbs N, Cullum N. Protease‐modulating matrix treatments for healing venous leg ulcers. Cochrane Database of Systematic Reviews 2016(12).

92. Wieland LS, Skoetz N, Pilkington K, Vempati R, D'Adamo CR, Berman BM. Yoga treatment for chronic non‐specific low back pain. Cochrane Database of Systematic Reviews 2017(1).

93. Wiffen PJ, Derry S, Bell RF, Rice ASC, Tölle TR, Phillips T, et al. Gabapentin for chronic neuropathic pain in adults. Cochrane Database of Systematic Reviews 2017(6).

94. Wilhelmus KR. Antiviral treatment and other therapeutic interventions for herpes simplex virus epithelial keratitis. Cochrane Database of Systematic Reviews 2015(1).

95. Williams MA, Srikesavan C, Heine PJ, Bruce J, Brosseau L, Hoxey‐Thomas N, et al. Exercise for rheumatoid arthritis of the hand. Cochrane Database of Systematic Reviews 2018(7).

96. Wilsdon TD, Whittle SL, Thynne TRJ, Mangoni AA. Methotrexate for psoriatic arthritis. Cochrane Database of Systematic Reviews 2019(1).

97. Yamato TP, Maher CG, Saragiotto BT, Hancock MJ, Ostelo R, Cabral CMN, et al. Pilates for low back pain. Cochrane Database of Systematic Reviews 2015(7).

98. Yue J, Dong BR, Yang M, Chen X, Wu T, Liu GJ. Linezolid versus vancomycin for skin and soft tissue infections. Cochrane Database of Systematic Reviews 2016(1).

99. Zhang L, Weizer JS, Musch DC. Perioperative medications for preventing temporarily increased intraocular pressure after laser trabeculoplasty. Cochrane Database of Systematic Reviews 2017(2).

100. Zhang ML, Hirunyachote P, Jampel H. Combined surgery versus cataract surgery alone for eyes with cataract and glaucoma. Cochrane Database of Systematic Reviews 2015(7).
